# NLRP3 inflammasome in digestive diseases: From mechanism to therapy

**DOI:** 10.3389/fimmu.2022.978190

**Published:** 2022-10-26

**Authors:** Rui Qiang, Yanbo Li, Xincan Dai, Wenliang Lv

**Affiliations:** Department of Infection, Guang'anmen Hospital, China Academy of Chinese Medical Sciences, Beijing, China

**Keywords:** digestive system diseases, NLRP3 inflammasome, small-molecule chemical drugs, biologics, phytochemicals

## Abstract

Digestive system diseases remain a formidable challenge to human health. NOD-like receptor family pyrin domain containing 3 (NLRP3) inflammasome is the most characteristic multimeric protein complex and is involved in a wide range of digestive diseases as intracellular innate immune sensors. It has emerged as a research hotspot in recent years. In this context, we provide a comprehensive review of NLRP3 inflammasome priming and activation in the pathogenesis of digestive diseases, including clinical and preclinical studies. Moreover, the scientific evidence of small‐molecule chemical drugs, biologics, and phytochemicals, which acts on different steps of the NLRP3 inflammasome, is reviewed. Above all, deep interrogation of the NLRP3 inflammasome is a better insight of the pathomechanism of digestive diseases. We believe that the NLRP3 inflammasome will hold promise as a novel valuable target and research direction for treating digestive disorders.

## 1 Introduction

To keep the internal environment in balance, the body takes the form of inflammation adaptively responding to stimuli that are noxious, like infection, injury, poisoning, pressure, and autoimmune reaction (1). An appropriate inflammatory response could remove damaged or dead cells from the body and facilitate the repair of tissues. If the endogenous danger signals are excessively activated, the inflammatory cytokines will be released. This process could adversely affect the host and make it prone to pathological damage. NOD-like receptor family pyrin domain containing 3 (NLRP3) inflammasome is an intracellular mediator, a sentinel of cells, which can sense changes in cellular homeostasis, respond to cellular stress, initiate inflammatory cascade reaction, and is in close relation to a diversity of illnesses (2–5).

Excessive initiation of the NLRP3 inflammasome, which is associated with the severity of digestive disease, has been found in patients and animal models’ blood and tissues, demonstrating that NLRP3 inflammasome plays a vital role in the development of digestive disorders. Moreover, NLRP3 inflammasome genes knock-down or knock-out are valid in reducing pathological symptoms (6–9). Accordingly, inhibiting the NLRP3 inflammasome signal may represent an emerging strategy suitable for treating digestive illnesses.

Taken together, this paper offers an integrated overview of priming and activation of the NLRP3 inflammasome, and its role in the pathogenesis of digestive system diseases in recent years, including clinical and experimental evidence. Furthermore, this article reviews that the scientific evidence of small‐molecule chemical drugs, biologics, and phytochemicals under investigation or in clinical settings, acts on the NLRP3 inflammasome signaling pathway. Deep discussion of NLRP3 inflammasome is aimed at a better understanding of digestive diseases and provides an opportunity to prevent and treat these diseases.

## 2 Structure and activation of the NLRP3 inflammasome

When body is stimulated, pattern recognition receptors (PRRs) in the innate immune system can recognize danger-associated molecular patterns (DAMPs) and pathogen-associated molecular patterns (PAMPs) and fight against pathogens by causing inflammatory response. PRRs of living organisms mainly include Nod-like roll receptors (NLRs), Toll-like receptors (TLRs), RIG-I like receptors (RLRs), and C-type Lectin receptors (CLRs). NLRP3 inflammasome, one of the most popular and well-studied members of the 22 different NLRs family members in humans (10, 11), is often composed of three domains: nod-like receptor protein (NLRP), apoptosis-associated speck-like proteins containing a CARD (ASC) and pro-caspase-1. Under normal conditions, NLRP3 is in a state of self-repression. When PAMPs or DAMPs appear, NLRP3 is released from a self-inhibited form. Then the oligomerization occurs and combines NLRP3 with ASC through the N-terminal Pyrin domain (PYD). ASC acts as a bridge between the NLRP3 and the pro-caspase-1, and activates pro-caspase-1 (12). Pro-caspase-1 is an inactive form of the proenzyme and participates in proinflammatory and pyroptosis process of cells (13). Activated caspase-1 turns pro-interleukin-1β (pro-IL-1β) and pro-interleukin-18 (pro-IL-18) to mature, biologically activates IL-1β and IL-18 (14, 15), and initiates an inflammatory cascade. Activated caspase-1 can also cut gasdermin-d (GSDMD), expose its N-terminal domain and aggregate on the cell membrane to form holes, resulting in continuous cell expansion, released contents, and facilitated pyroptosis. Eventually, inflammatory response and NLRP3 inflammasome reinforce each other, forming a vicious cycle which accelerates disease progression (16–18).

The activation of NLRP3 inflammasome consists of canonical and non-canonical ways. The canonical NLRP3 inflammasome activation requires two types of signals. The first signal is the priming signal. Stimulating factors can pass through important membrane surface receptors such as TLRs. All TLRs except TLR3 can activate myeloiddifferentiationfactor88 (MyD88) signaling pathway which activates nuclear transcription factor-κB (NF-κB) (19). NF-κB is a potent inflammatory activator that induces relatively high expression of NLRP3, pro-IL-1β, and pro-IL-18 and keeps them ready for activating. Conversely, without this step, the downstream expression would attenuate. Studies have disclosed that fas-associated with death domain protein (FADD), nucleotide-binding oligomerization domain 1/2 (NOD1/2), as well as caspase-8 were also involved in the priming of NLRP3 inflammasome (20–22). Secondly, the activation signal is needed. Conditions of activating NLRP3 inflammasome are varied, including (1): DAMPs or PAMPs induce K^+^ efflux by activating purinergic 2X7 receptor (P2X7R) on the cell surface or releasing adenosine triphosphate (ATP), so that NLRP3 inflammasome is triggered (23). Nevertheless, the K^+^ efflux is an important, but not a specific event in the NLRP3 inflammasome activation (24) (2). Stimulants (such as silica, asbestos, amyloid-β, alum) enter the cell through active transport, change the lysosomal membrane potential, interfere with its stability, and release lysoproteolytic enzymes, among which the cathepsin B may play a vital role in the activation of NLRP3 inflammasome (25). (3) Endogenous danger signals promote mitochondrial production of reactive oxygen species (ROS). Thioredoxin-interacting protein (TXNIP) separates from thioredoxin (TRX) and interacts with NLRP3 under ROS conditions, leading to NLRP3 inflammasome activation. These various upstream signaling pathways may be interrelated or independent and have been confirmed to trigger oligomerization of the NLRP3 protein complex (26).

**Figure 1:**
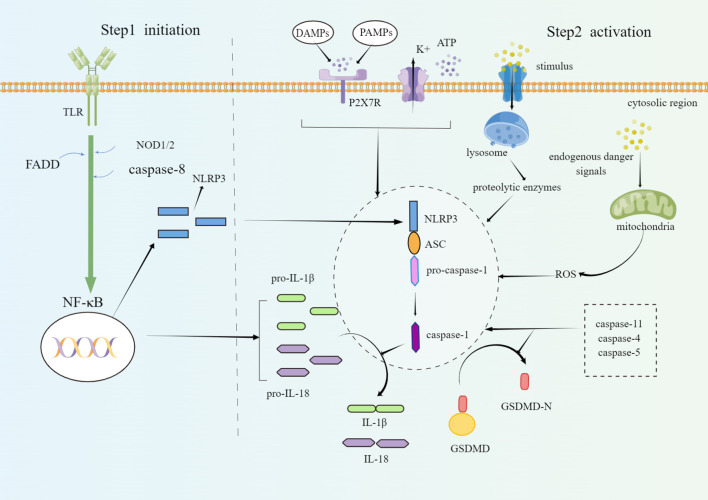
Schematic diagram of the molecular mechanism of NLRP3 inflammasome priming and activation. The priming steps of NLRP3 are regulated by TLR, FADD, caspase-8, and NOD1/2, which facilitate the activation of NF-κB and induce the activation of NLRP3. Canonical conditions of NLRP3 inflammasome oligomerization and activation include ATP, P2X7R, lysosomal damage, cathepsin release, K^+^ outflow, mitochondrial ROS damage, etc. On the one hand, activated caspase-1 cleaves GSDMD into a lipophilic N-terminal soluble in the cytoplasm and a hydrophilic C-terminal that can be embedded into the cell membrane. The GSDMD-N terminal domain will combine with the phospholipids on the cell membrane to form holes and induce pyroptosis. On the other hand, activated caspase-1 releases inflammatory cytokines such as IL-1β and IL-18. Non-canonical NLRP3 inflammasome activation is mediated by caspase-4, caspase-5, and caspase-11. By Figdraw.

The non-canonical NLRP3 inflammasome activation is mediated by caspase-11. Caspase-11 facilitates GSDMD activation and split, then mediates pyroptosis (17). Caspase-11 does not cleave interleukins but only leads to pyroptosis. The body also relies on caspase-4 and caspase-5 proteins, which have similar functions to caspase-11, to promote the non-canonical oligomerization and NLRP3 inflammasome activation (27). Both canonical and non-canonical NLRP3 inflammasome activation occur independently. However, non-canonical caspase-11 enhances canonical caspase-1 processing and IL-1β/IL-18 production by specific stimuli (e.g., cholera toxin or E. coli) (18) (**Figure 1**).

## 3 The role of NLRP3 inflammasome in the pathomechanism of digestive diseases

**Figure 2:**
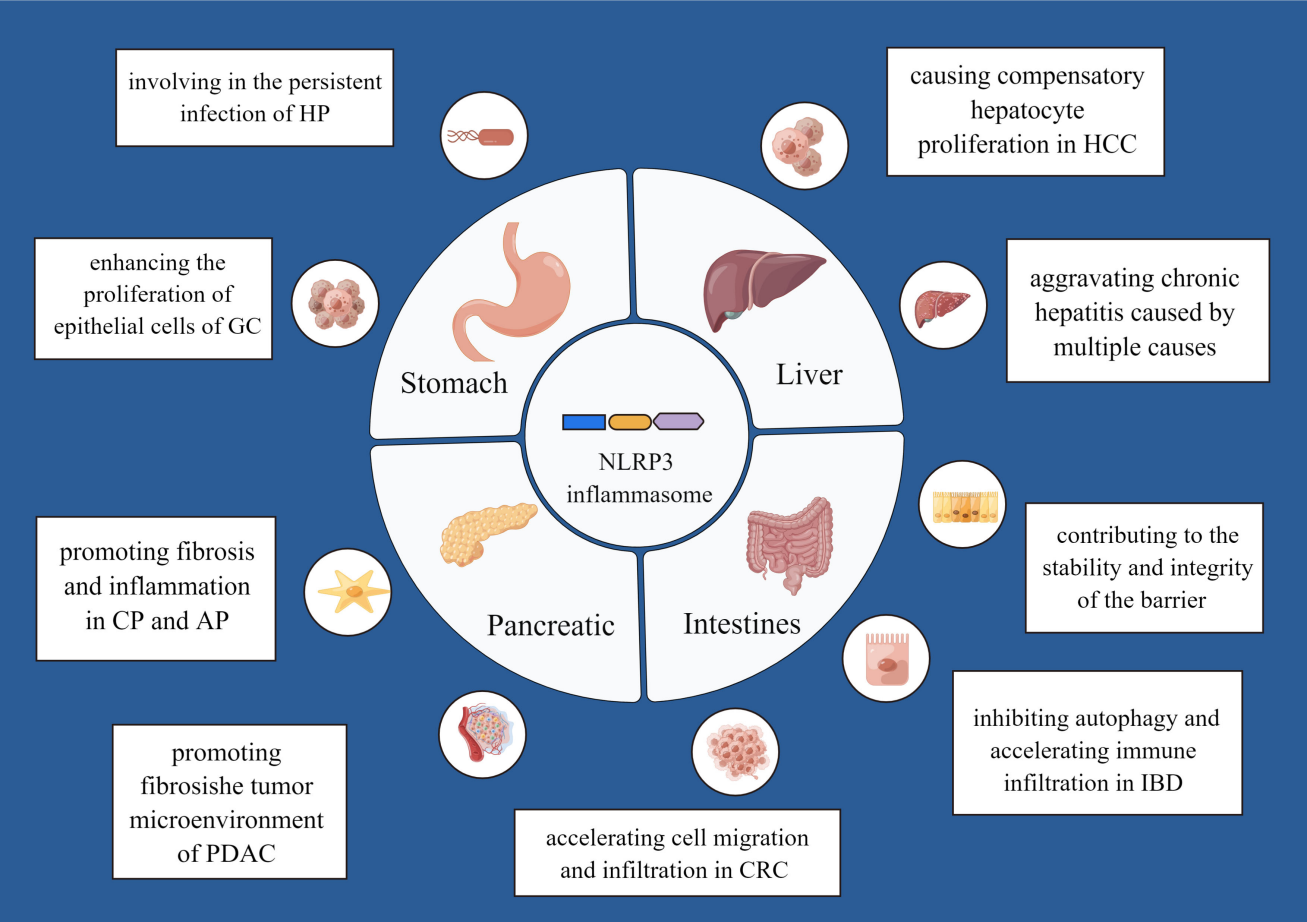
The mechanism of the NLRP3 inflammasome in various digestive diseases. By Figdraw.

Different digestive diseases may be caused by persistent inflammatory response. Studies in the field of clinical and preclinical research have demonstrated that the NLRP3 inflammasome may be associated with digestive disorders. We summarize current and prominent evidence to discuss in the following sections (**Figure 2**).

### 3.1 NLRP3 inflammasome and stomach disease

#### 3.1.1 Helicobacter pylori-related gastritis

Helicobacter pylori (HP) is the only harmful pathogenic bacterium ever found that can survive in human gastric mucosa. HP is listed as a class I carcinogenic factor by the World Health Organization (WHO) and belongs to infectious diseases (28). It was found that the content of NLRP3 and GSDMD were significantly higher in the gastric tissues of HP-infected patients (29). Experiments showed that the early stages of HP infection activate the NLRP3 inflammasome by targeting hsa-miR-223-3p and IL-10 (30). HP also provides a second signal required for the activation of the NLRP3 inflammasome, including K^+^ efflux and ROS production, which leading to a rise in IL-1β secretion (31). During HP infection, considerable mature IL-1β could cause atrophic gastritis and even cancer (32). Koch KN et al. reported that excessive activation of NLRP3 inflammasome keeps HP away from the immune system, persistently colonizes, and survive in the gastric niche (33). HP infectious mice models with NLRP3 knocked-down or knocked-out were protected against gastritis (34). These results indicate that HP infection can promote NLRP3 inflammasome activation which is involved in the persistent infection of HP. In other words, the NLRP3 inflammasome and HP infection promote each other and form a vicious circle.

#### 3.1.2 Gastric cancer

Gastric cancer (GC) is a malignant tumor that arises from the epithelial cells of gastric mucosa. It ranks fifth in terms of prevalence and third in terms of mortality around the world, with a poor prognosis (35). Persistent and chronic inflammation leads to the development of GC. NLRP3, as an essential inflammatory component, plays a crucial role in the progression of GC. The expression of NLRP3 in GC tissues exhibited a higher level than healthy tissues. Furthermore, redundant amount of ASC was found in most GC tissues in the Oncomine database and the The Cancer Genome Atlas (TCGA) database. It has been found that NLRP3 can enhance the uncontrolled proliferation of epithelial cells and the progression of GC. And when NLRP3 is inhibited, so does its carcinogenic influence *in vitro* and *in vivo* (36, 37). The NLRP3 inflammasome induces IL-1β production, and IL-1β can promote intestinal metaplasia, atypical hyperplasia, and proliferation of GC cells (38, 39). Therefore, NLRP3 by a combination of dependent and independent inflammasome pathways may stimulate GC development. Notably, the gene polymorphism of NLRP3 also impacts the pathogenesis of GC (40). These findings indicate that the NLRP3 inflammasome and its inflammatory products has a close relationship with GC development but more molecular pathways between the NLRP3 inflammasome and GC require further elucidation.

### 3.2 NLRP3 inflammasome and liver disease

#### 3.2.1 Viral hepatitis

Chronic infection with viral hepatitis affects half a billion individuals worldwide (41). Chronic hepatitis B (CHB) is a inflammatory liver illness caused by persistent infection of hepatitis B virus (HBV), which is a non-cytopathic double-stranded, hepatotropic DNA virus. Expression levels of NLRP3, ASC, and IL-1β in liver tissues from CHB patients were positively correlated with HBV-DNA concentration (42). It has been confirmed that NLRP3, IL-18, IL-1β, and caspase-1 were highly expressed in liver tissues of patients with HBV-related acute-on-chronic liver failure (HBV-ACLF). Patients with HBV-ACLF are likely to have impaired immune systems due to chronic inflammation (43). Similarly, hepatitis B core antigen promoted the expression of NLRP3 inflammasome and IL-1β (44). These results might suggest that persistent infection with HBV makes the NLRP3 signaling pathway activated, which is involved in the inflammatory response and injury of liver tissues by mediating cytokines such as IL-1β, and IL-18. Whereas the specific mechanism of the NLRP3 inflammasome activation infected by HBV has not been well understood. Hepatitis virus C (HCV), as a hepatotropic and enveloped virus, carries a positive-sense single-stranded RNA genome, and chronically infects around 3% of people worldwide (45). Negash et al. showed that the serum levels of IL-1β were significantly increased in patients with chronic hepatitis C (CHC). Furthermore, they showed that HCV RNA triggers MyD88-mediated TLR7 signaling and the NLRP3 inflammasome pathway to induce IL-1β production (46). Once infected with HCV, ASC is recruited to NLRP3 and dissociates from Golgi-resident protein immunity-related GTPase M (IRGM), causing Golgi fragmentation. This process enhances the replication of HCV and promotes chronic inflammation of the liver (47). Ramachandran A et al. demonstrated that the NLRP3 inflammasome, assembled and activated in HCV-infected liver cells and regulated by post-translational modifications, was critical in the pathogenesis of CHC (48). A study found that inoculation with infectious Hepatitis E virus (HEV) particles robustly triggered NLRP3 inflammasome activation in primary macrophages and macrophage cell lines. Interestingly, inflammasome activation antagonizes interferon response to facilitate HEV replication in macrophages (49). Taken together, strategies to inhibit the NLRP3 inflammasome or its inflammatory cytokines provide therapeutic choice to alleviate the liver inflammation besides antiviral agents.

#### 3.2.2 Non-alcoholic fatty liver disease

Non-alcoholic fatty liver disease (NAFLD) is a progressive hepatic disease with ectopic fat accumulation in the liver caused by harmful factors, other than alcohol. The prevalence of NAFLD varies between 19% and 33% in epidemiological studies and remains a challenging issue for human health (50). Multiple studies have demonstrated that the NLRP3 inflammasome is a pivotal contributor to the amplification of hepatic inflammation, immune cell activation, and hepatocyte damage (51). Caspase-1, the activated form after NLRP3 inflammasome activation, is present in the serum of patients with NAFLD, and its levels are closely correlated with disease severity (52). A high level of NLRP3 and caspase-1 is activated in macrophages in response to an excessive accumulation of lipids in dead hepatocytes (53). The NLRP3 inflammasome is associated with nonalcoholic steatohepatitis (NASH) which is a severe procedure in the advancement of NAFLD. NASH is distinguished from the NAFLD by the additional presence of features of hepatocellular injury with or without fibrosis (8). There is increasing evidence that NLRP3-mediated pyroptosis is key to the progression of simple steatosis to NASH (17, 54). Pyroptosis leads the NLRP3 inflammasome to the extracellular fluid. From there, the NLRP3 inflammasome is taken up by other cells, thus mediating inflammation and fibrosis (52). A study has confirmed that inhibition of pyroptosis can alleviate the inflammatory response of liver tissue by targeting the NLRP3 inflammasome (55). There is evidence that NLRP3 inflammasome plays a direct role during the development of NASH/NAFLD in mice models. It was found that mice with NLRP3 inflammasome loss of function are protected from diet-induced steatohepatitis (56). NLRP3 inflammasome gain of function leads to the early and severe onset of diet-induced steatohepatitis in mice (9). Inhibiting the NLRP3 inflammasome particularly alleviated inflammation, lipid accumulation, and fibrosis in NASH/NAFLD (5, 55, 57–59). Whereas there is also evidence that NLRP3 produces a hepatoprotective effect in NASH/NAFLD models. Jorge Henao-Mejia et al. observed that NLRP3-deficient aggravates NASH, which makes serum alanine transaminase(ALT), aspartate transaminase (AST), and NAFLD activity inflammation scores increase (60). The NAFLD mouse model lack of NLRP3 showed higher triglyceride content, histological score of liver injury and greater adipose tissue inflammation (61). Different metabolic processes, tissues, and mouse models could account for the above conflicting findings (61).

#### 3.2.3 Alcoholic liver disease

Alcoholic liver disease (ALD) is a kind of liver disease caused by long-term heavy drinking. The initial stage usually presents with alcoholic hepatitis and hepatic fibrosis, with the consequent risk of developing into cirrhosis or liver cancer. Severe alcoholism could induce extensive necrosis of liver cells and even liver failure. A preliminary study showed that the messenger RNA (mRNA) levels of the components of the NLRP3 inflammasome (IL-1β, IL-18, and caspase 1) tended to be greater in patients with severe liver damage (alcoholic hepatitis histological score≥5) than in patients with milder liver damage (62). Studies have demonstrated that long-term intake of alcohol facilitated liver damage and promoted the expression of IL-1β, caspase-1, ASC, and NLRP3 in wild-type (WT) mice (63). As for the mechanism of NLRP3 activation during ALD, it has been elucidated that the metabolic disorders of uric acid and ATP induced by alcohol could lead to mitochondrial damage, and the activation of NLRP3 inflammasome. Additionally, alcohol decreases miR-148a expression in hepatocytes through forkhead box protein O1 (FoxO1), facilitates TXNIP overexpression and activates the NLRP3 inflammasome, which induces hepatocyte pyroptosis and aggravates ALD (64). Recent studies have demonstrated that ethanol-induced NLRP3 activation was primarily caused by the downregulation of aryl hydrocarbon receptors and the activation of TXNIP in human macrophages, both of which were caused by oxidative stress (65). In summary, alcohol induces NLRP3 inflammasome activation through various pathways, and inhibition of NLRP3 inflammasome activation is capable of abating ALD (66, 67). On the contrary, it is also found that NLRP3 deficiency mice have more severe hepatic damage with higher levels of ALT and IL-18 levels (68). These contradictory outcomes could be explicated by various experimental conditions and approaches.

#### 3.2.4 Drug-induced liver injury

Drug-induced liver injury (DILI) refers to liver injury caused by various prescription or non-prescription drugs and their metabolites and excipients. Clinical manifestations of DILI are acute hepatitis, cholestasis, or jaundice. It develops independently of drug dose, route, or duration of administration (69). The pathogenesis of DILI is complex and different. If left untreated, liver failure may develop with high mortality. Rifampicin (RIF) and isoniazid (INH) are the classical anti-tuberculosis medicines (70, 71). However, INH and RIF can significantly change the structure of normal liver tissues and cause inflammation, leading liver damage or hepatotoxicity. Many researches had confirmed that the NLRP3 inflammasome was required for INH and RIF-induced liver injuries (5, 72). Moreover, the knock-down of NLRP3 with small interfering RNA (siRNA) dramatically changed the effects of INH and RIF on hepatocytes (73). It was found that the NLRP3 inflammasome was also the key to acetaminophen(APAP)-induced DILI (74). APAP-induced DILI is protected by aspirin and benzyl alcohol, which inhibit the activation of NLRP3 inflammasomes and neutrophil infiltration (75, 76). However, the role of the NLRP3 inflammasome in APAP-induced liver injury is disputed. Studies have described no protective effect if inflammasome components, such as NLRP3, caspase-1, ASC, are deficient in APAP-induced liver injury. The reason for these opposite results remains unclear (77).

#### 3.2.5 Cholestatic liver injury

Cholestasis, characterized by bile secretion disorder and excessive bile acid (BA) accumulation in the liver, is clinically associated with a variety of liver diseases, such as progressive familial intrahepatic cholestasis, primary biliary cirrhosis (PBC), and primary sclerosing cholangitis (PSC) etc. An injury to the liver caused by cholestatic disease results in the death of cells followed by inflammation and fibrosis. There is a correlation between NLRP3 activation and chronic cholestasis in humans and mice (78). Expression of NLRP3 was significantly elevated in bone marrow-derived monocytes/macrophages of bile duct ligation (BDL) mice (79). Twenty-eight days after BDL, bridging fibrosis was observed in WT mice instead of mice with NLRP3 knocked out. Lack of NLRP3 expression attenuated liver injury and fibrosis after acute and chronic BDL. The NLRP3 small molecule inhibitor MCC950 could reduce BDL-induced disease progression in WT mice (80) by reducing the production of the pro-inflammatory cytokines IL-1β and IL-18 and inhibiting neutrophil infiltration and hepatic cell death (72), emphasizing the importance of the NLRP3 inflammasome in cholestatic liver injury onset and progression.

#### 3.2.6 Autoimmune hepatitis

Autoimmune Hepatitis (AIH) is a kind of autoimmune diseases. In AIH, loss of tolerance against liver autoantigens causes elevated serum aminotransferase levels, presence of non-organ-specific autoantibodies with unknown function, hyperglobulinemia, progressive destruction of the hepatic parenchyma, and development of hepatic fibrosis (81). Recent studies have indicated that the NLRP3 inflammasome drives pathogenesis of concanavalin-A (ConA)-induced hepatitis. Liver pathology was related to elevated levels of NLRP3, IL-1β, caspase-1 and pyroptosis-mediated cell death (82). Moreover, ConA could induce activation of the NLRP3, caspase-1 and IL-1β production in macrophages *in vitro*. IL-1β levels were elevated in AIH patients and correlated with aggravation of hepatitis (83). NLRP3 inflammasome also participates in Trichloroethene (TCE) -induced AIH (84). Using an interleukin-1 receptor (IL-1R) antagonist could suppress hepatic inflammation of AIH by diminishing NLRP3 inflammasome activation. The miR-223 negatively regulates NLRP3 expression (85). The application of exosomes containing miR-223 decreases NLRP3 and caspase-1 expression and promotes AIH to abate (86). Collectively, these findings demonstrate that the NLRP3 inflammasome signaling pathway plays a crucial role in the initiation, progression, and development of AIH, and inhibition of the NLRP3 inflammasome signaling pathway also offers a new and appropriate therapeutic strategy for the treatment of AIH.

#### 3.2.7 Hepatic fibrosis

Hepatic fibrosis (HF) is vigorous as well as a reversible process of wound healing, where quiescent hepatic stellate cells (HSCs) proliferate and transdifferentiate into myofibroblasts which are responsible for depositing extracellular matrix (ECM) proteins, leading to tissue scarring. HF is a crucial step and inevitable stage in transforming various chronic liver diseases into cirrhosis and liver cancer (87). In the progression of HF, the aberrant regulation of inflammation is a prominent driving factor. NLRP3 inflammasome plays an essential role in the fibrosis process of liver tissues (88). Inzaugarat, Maria Eugenia et al. found that NLRP3 inflammasome activated in HSCs directly contributes to HF development without the presence of inflammatory infiltrates (89). Transgenic mice expressing constitutively active NLRP3 showed severe hepatocyte pyroptosis, inflammation, and fibrosis (90). It also has been found that the NLRP3 inflammasome participates in HF caused by schistosomiasis (91). Deficiency or inhibition of NLRP3 inflammasome can improve HF, hepatic inadequacy, liver inflammation, granuloma, and hepatosplenomegaly caused by schistosomiasis (7, 92). Many factors can activate the NLRP3 inflammasome, leading to the secretion of IL-1β and IL-18, and induce HF (52). For example, Androgen, an activator of the NLRP3 inflammasome, can aggravate liver damage in carbon tetrachloride (CCL_4_)-induced HF mice model (93). Galectin-3 is a multifunctional glycoprotein that promotes infiltration by mononuclear cells by enhancing the production of NLRP3 inflammasome and IL-1β, which promotes granuloma and HF (94). Correspondingly, many factors can delay or reverse HF by inhibiting NLRP3 inflammasome activation (95, 96). Overall, these findings strongly indicate that NLRP3 inflammasome plays a key role in HF, the mechanisms by which the NLRP3 inflammasome regulates fibrogenesis are not well understood. Further, it remains unclear whether IL-18 promotes or inhibits fibrogenesis (97) and more research is required.

#### 3.2.8 Hepatocellular carcinoma

Hepatocellular carcinoma (HCC) is the most common primary malignant tumor of the liver. It frequently develops in the context of chronic hepatitis and cirrhosis and is the third leading cause of cancer death worldwide (98). The presence of a chronically inflammatory microenvironment is one of the factors that can promote tumorigenesis and metastasis (99). The overexpressed NLRP3 in HCC indicated worse overall survival. In HCC, inhibition of NLRP3 promotes the killing effect of T cells to cancer cells by repressing the expression of immune checkpoints (100). A study found that a deletion of NLRP3 suppresses cancer development and metastasis of HCC cells *in vitro* and *in vivo* (97). However, it was also demonstrated that a deficiency in NLRP3 inflammasome expression is involved in HCC progression, which suggests that the NLRP3 inflammasome has a protective role against the development of HCC (101). These opposite conclusions may be due to the different functions of the NLRP3 inflammasome. It is important to note that the NLRP3 inflammasome represents the first line of defense against pathogenic microbes within the innate immune system. When the body is exposed to a pathogenic insult, the NLRP3 inflammasome acts as a guard and is assembled and activated to elicit hepatic inflammation and return the system to homeostasis. However, chronic persistent inflammation and inflammasome abnormal activation induces massive cell death, and NLRP3’s homeostatic threshold could be exceeded, then causing compensatory hepatocyte proliferation and HCC.

### 3.3 NLRP3 inflammasome and pancreatic disease

#### 3.3.1 Chronic pancreatitis

Chronic pancreatitis (CP) is a syndrome caused by genetic and environmental factors (102) and characterized by inflammatory cell infiltration, progressive organ atrophy, and disorder of collagen deposition (103). There is increasing evidence that NLRP3 inflammasome plays a vital role in the inflammatory response of pancreatic tissues. Zhang G et al. observed that NLRP3 activation mediates caspase-1 activation and promotes the activation of pro-IL-1β and pro-IL-18, which are key pro-inflammatory cytokines in the pathological mechanism of CP (104). Through P2X7R inhibitor, Zhang GX inhibited the secretion of IL-1β and IL-18 which were dependent on NLRP3 to reduce chronic pancreatic inflammation and fibrosis, which confirms the pivotal role of the NLRP3 pathway in the occurrence and development of pancreatitis (105). Furthermore, NLRP3 inflammasome also participates in the fibrotic process of CP (106). Abnormal activation of pancreatic stellate cells (PSCs) releases a vast amount of ECM containing type I collagen (Col I), type III collagen (Col III), and fibronectin (FN), which plays a significant role in the extensive fibrosis of pancreatic tissues (107). Li CX et al. verified the causality between NLRP3 inflammasome and the abnormal activation of PSCs. They observed that the NLRP3 inflammasome is directly involved in activating PSCs *in vivo* and *in vitro*. Inhibiting NLRP3 suppresses the activation of PSCs and ECM deposition, thus alleviating pancreatic fibrosis (108). In conclusion, the above data reveals that NLRP3 inflammasome has a pro-inflammatory and pro-fibrogenic role in CP.

#### 3.3.2 Acute pancreatitis

Acute pancreatitis (AP), one of the fatal diseases of the gastrointestinal tract, is a kind of aseptic inflammation characterized by pancreatic enzyme activation and partial pancreatic inflammatory reaction. Studies have confirmed that while pancreatic inflammation may be initially triggered by intra-acinar events such as trypsinogen activation, it ultimately depends on the subsequent immune responses induced by the activation of inflammasome of the innate immune system. Neutrophils and macrophages from pancreatic acinar cells (PACs) recognize PAMPs and DAMPs in the damaged pancreatic tissues of AP (12), increase NF-κB and NLRP3 inflammasome expression and inducie the release of downstream inflammatory factors, which eventually promote the damage and inflammation of pancreatic tissues (109). IL-1β and IL-18 have been recognized as markers of severity of AP (110). In contrast to other cytokines, IL-1β and IL-18 are synthesized as precursor proteins and require cleavage in order to be biologically active. A key component of this process is the NLRP3 inflammasome. It has been demonstrated by Hoque and Mehal that the NLRP3 inflammasome is substantially activated during AP, and that components of this inflammasome are crucial for the development of total pancreatic injury. An experimental model of cerulein-induced AP in mice showed significant reductions in edema and systemic inflammation when caspase-1, ASC, and NLRP3 were not present (12). Wang, J, etc. reported that the activation of NLRP3 inflammasome and promotion of caspase-1-induced pyroptosis aggravate AP (111). Overactivation of NLRP3 inflammasome had also been found to contribute to the pathomechanism of AP by exacerbating intestinal dysfunctions (112). Recently, a meta-analysis demonstrated that the inhibition of NLRP3 inflammasomes significantly decreased pancreatic histopathological scores, serum amylase levels and lipase levels as well as circulating levels of inflammatory cytokines and reduced the severity of acute lung injury and acute intestinal injury as a result of AP. It has been recognized that the NLRP3 inflammasome plays a significant role in the pathogenesis and complications of AP (113).

#### 3.3.3 Severe acute pancreatitis

Approximately 20% of AP patients suffer from acute exacerbation, pancreatic necrosis, systemic inflammatory response, and multiple organ failure, which candevelop into severe acute pancreatitis (SAP) (114). SAP is a common acute disease in the digestive system, with the characteristic of rapid progression, which might cause multi-organ complications. Previous studies have confirmed that the fast production and release of a large number of inflammatory cytokines damage the pancreas and other organs (115). This process plays a crucial role in the pathological process of SAP. Sendler M et al. observed that mRNA content of NLRP3 and protein content of NLRP3 inflammasome were significantly increased in tissues of SAP mice, and deletion of NLRP3 reduced neutrophil maturation and macrophage infiltration, thereby reducing immune/inflammatory responses of the body (116). A significant reduction in systemic inflammatory response syndrome and compensatory anti-inflammatory response syndrome was observed in mice with severe pancreatitis that were inhibited by NLRP3 (116). By comparing the pathological tissues of (NLRP3 +/+) and (NLRP3 -/-) WT mice, it was found that knock-out NLRP3 plays a crucial role in alleviating SAP-related inflammation and lung injury. Similar results were confirmed in SAP mice models treated with NLRP3 inhibitors (117). Overall, the above findings suggest that SAP and its complications were characterized by activation of NLRP3 inflammasome. Most importantly, these results provide a potential molecular target for treating pancreatic disease.

#### 3.3.4 Pancreatic ductal adenocarcinoma

A tumour of the digestive system that is highly malignant and highly invasive is pancreatic cancer. Pancreatic ductal adenocarcinoma (PDAC) is the most common type of pancreatic cancer, accounting for about 90% of pancreatic cancer (118). Pancreatic ductal adenocarcinoma (PDAC) is a substantial global threat to human health. Previous studies have confirmed that inflammatory injury is a significant risk factor for PDAC. Romero JM et al. observed up-regulation of NLRP3 inflammasome pathway by RNA sequencing and whole-genome sequencing in cancer tissues of patients with primary resected PDAC and PDAC liver metastases (119). Similar results were obtained in experiments, in which up-regulation of NLRP3 inflammasome expression was observed in platelets of mice models with primary PDAC (120). Daley D et al. further verified the indispensable role of the NLRP3 inflammasome in the pathological mechanism of PDAC by observing the protective effect of pharmacological inhibition or deletion of NLRP3, ASC, and caspase-1 in PDAC mice models (121). Findings identify a new modality for immune evasion in PDAC that depends on IL-1β production by tumor cells through TLR4-NLRP3 inflammasome activation. IL-1β is an essential component of PDAC’s immune toleranc (122). NLRP3 plays a significant role in establishing the microenvironment surrounding the PDAC tumor by modulating the expression of IL-1β (123).

### 3.4 NLRP3 inflammasome and intestines disease

#### 3.4.1 Inflammatory bowel disease

Inflammatory bowel disease (IBD) is a chronic, recurrent gastrointestinal disorder that does not present structural or biochemical abnormalities, and it is associated with abdominal pain, distention of the abdomen, changes in the bowel habits, and changes in stool characteristics. The etiology is caused by environmental factors, infectious agents, and genetic susceptibility, including ulcerative colitis (UC) and crohn’s disease (CD). Clinical evidence uncovered that upregulation of NLRP3 and IL-1β were observed in UC patients (124) and CD patients (125). It was also shown that NLRP3 inflammasome and downstream effector expression including IL-1β are increased in inflamed mucosa of IBD patients and correlate with disease activity. Inflammasome gene expression increased with the abundance of immature intestinal macrophages (126). Research demonstrates that NLRP3 acts as a molecular switch by shifting local immune cells toward an inflammatory phenotype *via* IL-1β (122). Experiments observed the effects of NLRP3 deletion or inhibition or blocking oligomerization on the development of intestinal inflammation and concluded that the overactivation of NLRP3 inflammasome and its major cytokines promotes the development of IBD (127–131). It was also found that mice genetically deficient in miR-223 display markedly exacerbated experimental colitis, as indicated by increased immune infiltration (neutrophils and monocytes), hyperactivated NLRP3, and IL-1β release. Meanwhile, nanoparticle delivery of miR-223 mimetics can constrain the level of NLRP3 activation, provides an early break, limiting cytokine-mediated immune disequilibrium, and attenuate experimental IBD (4). There is, however, debate regarding whether the NLRP3 inflammasome contributes to IBD in a beneficial or pathogenic manner. A number of studies have shown that the NLRP3 inflammasome plays a key role in regulating mucosal immune responses as well as intestinal homeostasis (61). NLRP3-induced IL-18 is required for the proliferation of intestinal endothelial cells. Hence, it has been speculated that the appropriate activation of the NLRP3 inflammasome in the intestinal epithelial cells contributes to the maintenance of internal environmental stability and integrity of the barrier. This discovery could be related to the compensatory response of the NLRP3 inflammasome stimulating the proliferation of epithelial cells (132). It has identified that early activation of NLRP3 in intestinal epithelial cells limits pathogen colonization and prevents subsequent intestinal inflammation (133). Reports are suggesting that IL-1β and IL-18 induced by the NLRP3 inflammasome confer protection against colitis and colitis-associated tumorigenesis (134–136). Moreover, hyperactive NLRP3 maintains gut homeostasis by inducing regulatory T cells (Tregs) (137). Studies also found the deficiency of NLRP3 inflammasome composition increases susceptibility to experimental colitis in mice (134, 138, 139). NLRP3 also plays a protective role in the probiotic-based therapy of colitis (140). These controversial outcomes may be due to the experimental protocols, different facilities, variable effects of microbiota, and the genetic background of mice. IBD therapeutic approaches will be inspired by further studies on the molecular regulation of the NLRP3 inflammasome activity during inflammation.

More and more evidence focus on autophagy and NLRP3 inflammasome interaction in the development of IBD in its extended form. Autophagy has an excellent negative regulatory effect on the activation of NLRP3 inflammasome, including removal of the sources of endogenous activation of NLRP3 inflammasome, ROS inhibition, removal of damaged mitochondria, and selective degradation of inflammasome components (14, 141). NLRP3 inflammasome signal also negatively regulates autophagy. NLRP3 may be a binding partner of the inhibitors of autophagy. In IBD mice models, NLRP3 binds to mTOR (an autophagy inhibitor) to promote the phosphorylation of mTOR and inhibite autophagy (142). Quach C et al. found NLRP3 inflammation hyperactivation in an autophagy inhibition mouse model. NRLP3 inflammasome inhibition also might be a strategy that promotes the activation of autophagy in an efficient manner. Collectively, the NLRP3 inflammasome activation dynamically interacts with autophagy during the development of intestinal inflammation (143), and the balance between autophagy and the NLRP3 inflammasome helps to maintain the homeostasis of the host. These findings also raised the possibility of using NLRP3 inhibitors that induce autophagy or autophagy activators that inhibit inflammasome as potential therapeutic strategies for IBD treatment.

To further explore the pathological mechanism of IBD from the perspective of gene polymorphism, the role of NLRP3 inflammasome-related genes in IBD has been researched. ZhouL et al. found that NLRP3 is encoded by RS772009059 (R779C) in 3 patients with early-onset IBD. In dextran sulphate sodium (DSS)-induced models of acute colitis, R779C promotes the activation and apoptosis of the NLRP3 inflammasome in macrophages and is positively correlated with the incidence of severe diseases (144). Studies indicate that some genotypes of the NLRP3 inflammasome may be associated with the affectability of patients with IBD and could represent biomarkers for the evaluation of IBD severity. A Swiss cohort study found that three NLRP3-related single nucleotide polymorphisms (RS10733113, RS55646866, rs4353135) were negatively related to CD (145). Moreover, some negative results were found (146). In short, the gene polymorphism of the NLRP3 inflammasome will provide new insights into IBD pathogenesis. Different NLRP3 gene therapy methods are waiting to be developed in the future. But the role of gene polymorphism of NLRP3 inflammasome in IBD has not yet been fully discovered. More research is needed in the future.

#### 3.4.2 Colorectal cancer

Colorectal cancer (CRC) is the third common malignancy and the second common cause of death from malignancies (147). Chronic gut inflammation is a critically predisposing factor for the development of CRC. Shi F et al. observed that NLRP3 expression is elevated in human tissues of CRC (100 cases) and mice models of colorectal adenocarcinoma. The elevated levels of NLRP3 are correlated with distant metastasis, vascular infiltration, and positive lymph nodes. In addition, the survival analysis of kaplan-Meier showed that high expression of NLRP3 was associated with a lower 5-year survival rate and lower 10-year survival rate (148). These results might elucidate that NLRP3 is an independent risk factor for CRC prognosis. Similarly, Marandi Y et al. compared the cancer tissues and adjacent normal tissues of 43 patients with CRC and concluded that the development of CRC was related to the NLRP3 inflammasome activation (149). NLRP3 activation was also found to accelerate the rate of cell migration of CRC. Blocking the NLRP3 signaling suppressed CRC cell migration *in vitro*, and metastatic ability *in vivo* (150). The NLRP3 inflammasome regulates the activation of caspase-1. Activation of caspase-1 enhances the secretion of IL-1β and IL-18. IL-1β and IL-18 lead to the infiltration of more immune cell, result in the generation and maintain an inflammatory microenvironment of CRC (151). Interestingly, the pro-inflammatory and carcinogenic effect of dietary cholesterol in CRC (152) and the carcinogenic effects of porphyromonas gingivalis in CRC are also related to NLRP3 inflammasome overactivation (153). Whereas the inflammasome complex is also a protectant in the intestinal epithelium. In research, NLRP3 deficient mice showed an increased diarrhea, rectal bleeding and mortality (135). The anti-tumor effect of IL-18 blocks tumors development as well as inhibits angiogenesis and may induce epithelial cell recovery (136). Furthermore, IL-18 can reduce cell proliferation in the intestinal epithelium at the tumor zone in the colitis remission phase (154). There are studies revealed that protective effect of pyroptosis mediated by NLRP3 on CRC. Tang Z et al. confirmed *in vivo* and *in vitro* experiments that NLRP3 inflammasome mediated pyroptosis could inhibit the proliferation, migration, and invasion of CRC cells (155). NLRP3 inflammasome has also been reported to enhance NK cell tumoricidal function mediated by IL-18 by inhibiting CRC metastatic growth (156). It has been found that in the intestinal mucosa with high expression of antigen, inflammation with low levels is indispensable for the harmony between the immune system and the microbiota and helps to maintain homeostasis (157). To sum up, controversy remains and some people believe that the NLRP3 inflammasome is a double-edged sword in the development of CRC (158, 159). While long-term effects of the NLRP3 inflammasome activation, and inflammatory reaction are associated with poor outcomes, mild inflammation may be beneficial.

### 3.5 Discussions

The discovery of NLRP3 inflammasomes has enriched our knowledge of the pathogenesis of multiple digestive diseases. The NLRP3 has emerged as the most versatile and well-characterized inflammasome. Undoubtedly, as tremendous advances in NLRP3 inflammasome continues, the possibility of NLRP3 inflammasome becoming a therapeutic target for the treatment of diseases is advanced. However, some mechanisms of NLRP3 inflammasome are still incompletely characterized, and even remain controversial and conflicting. We suggest that a primary role of NLRP3 is to sense noxious stimuli that accumulate, with NLRP3 driving inflammation to facilitate their clearance. These factors might accumulate during normal tissue damage, and the NLRP3 response would be self-limiting as the clearance succeeds. However, with more and more stimuli, NLRP3 becomes pathologic accumulation and leads to diseases. Most studies have also focused on the role of the canonical NLRP3 inflammasome pathway and its downstream products, ignoring other possible pathogenic mechanisms. The purpose of further investigation might be to elucidate the function of non-canonical NLRP3 signaling, other inflammasomes such as NLR family, pyrin domain containing 1 (NLRP1) and absent in melanoma 2 (AIM2) in digestive disorders, and the activation of the NLRP3 inflammasome in the complex mechanisms of organ-to-organ communication.

## 4 Treatment strategy

**Figure 3:**
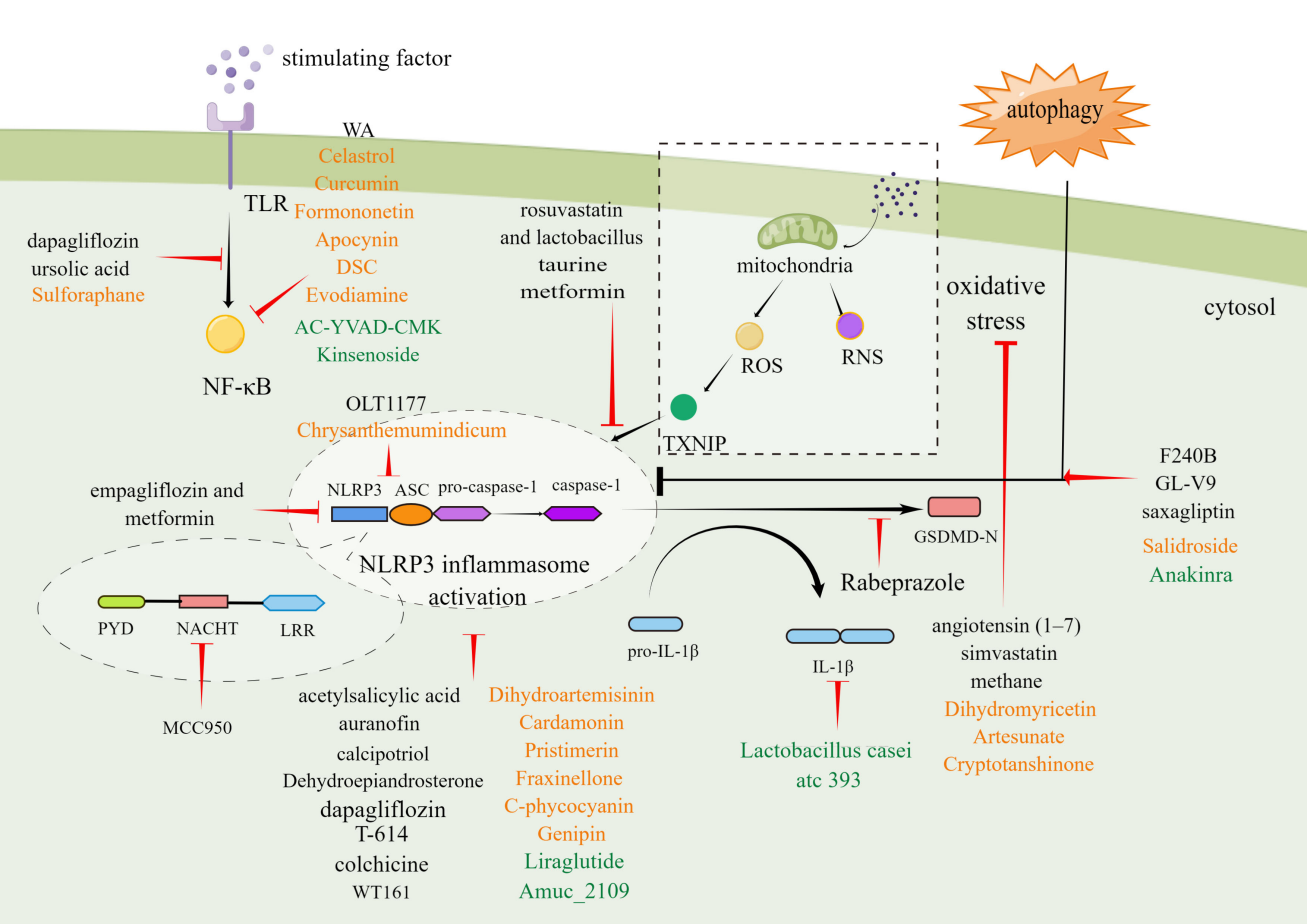
Molecular mechanism of small-molecule chemical drugs, biologics, and phytochemicals treat digestive diseases by inhibiting different steps in the NLRP3 inflammasome signaling pathway. Black font represents small-molecule drugs. Orange font represents phytochemicals. Green font represents biologics. By Figdraw.

As indicated by various published reports, pharmacological inhibition of the different steps underlying NLRP3 inflammasome activation may be a suitable strategy for treating digestive system disorders. The article details in-depth studies of small molecule chemicals, biologicals, and phytochemicals, focus on their mode of action and therapeutic potential, and summarize in **Tables 1**–**4** and **Figure 3**.

**Table 1:** Small‐molecule chemical drugs able to inhibit NLRP3 activation in animal models of digestive diseases.

Drugs	Molecular mechanisms	Experimental model	Reference	Clinical trials
MCC950	Modifying the active conformation of NLRP3 Affecting the hydrolysis of ATP	Pancreatic cancer Liver injury and fibrosis UC	(80, 160–162)	null
OLT1177	Preventing NLRP3 aggregation with ASC	DSS-induced colitis	(163)	null
WT161	HDAC6 inhibitor	IBD	(164)	null
	Blocking NLRP3 inflammasome activation, disrupting ASC speck formation, and decreasing the expression of NLRP3			
Withaferin A (WA)	Inhibition of NF-κB signaling pathway	Pancreatitis	(165)	null
F240B	Induction of SIRT1-dependent autophagy	Peritonitis	(166)	null
	Inhibiting ASC oligomerization and pro-IL-1β expression			
GL-V9	Trigger of autophagy to degrade NLRP3 inflammasome	Liver cancer	(167–169)	null
		Colorectal cancer		
Iguratimod (T-614)	Inhibition of NLRP3 inflammasome activation	SAP	(170)	null
Methane	Inhibition of TLR4/NF-κB/NLRP3 signaling pathway	Cholestatic liver injury	(171)	null
Angiotensin- (1–7)	Inhibition of Ang II-mediated ROS	HF	(172)	null

**Table 2:** Small‐molecule chemical drugs currently approved for clinical use and endowed with the ability to inhibit NLRP3 activation.

Drugs	Molecular mechanisms	Experimental model	Reference	clinical trials
Calcipotriol	Activation of Yes-associated protein	Cholestatic liver Injury and fibrosis	(173)	null
Dehydroepiandrosterone	Inducing autophagy *via* GPER activation inhibited ERK Inhibition of NF-κB signaling pathway	Colitis	(174)	NCT00106314
Colchicine	Inhibition of NLRP3 inflammasome activation	Intestinal damage	(175)	null
Dapagliflozin	Inhibition of NF-κB/AMPK signaling pathway	Ulcerative colitis (UC)	(176, 177)	null
	Inhibition of NLRP3/caspase-1 signaling pathway	Steatohepatitis		
Ursolic acid	Inhibition of NF-κB signaling pathway	Gastric cancer (GC)	(37, 178)	null
		HF		
Acetylsalicylic acid	Inhibition of assembly and activation of NLRP3 inflammatory	Alcohol-and atorvastatin-induced hepatotoxic	(179)	NCT00898950
				NCT00272311
				NCT01250340
Saxagliptin	Activation of AMPK/mTOR-driven autophagy	Gastric mucosal damage	(180)	null
Rabeprazole	Suppression of Pyroptosis executed by GSDMD	HP infection	(29)	NCT02490839
				NCT02483715
				NCT01643785 etc
Metformin	ROS suppression *via* TXNIP-NLRP3-GSDMD pathway	Intestinal ischemia-reperfusion injury	(181)	NCT04750135
Simvastatin	Increase in antioxidant level	UC	(182)	null
Empagliflozin combined with Metformin	Interference With the AMPKα/mTOR/NLRP3 signaling	UC	(183)	null
Auranofin	Inhibition of NLRP3 inflammasome	NAFLD	(59)	null
Taurine	Inhibition of TXNIP/NLRP3 signaling pathway	HF in schistosomiasis	(7)	null
Rosuvastatin combined with Lactobacillus	Suppression the TXNIP/NLRP3 Interaction	UC	(184)	NCT04883840 (Rosuvastatin)

**Table 3:** Biologics able to inhibit NLRP3 inflammasome in digestive diseases.

Drugs	NLRP3 signaling molecular target	Experimental model	Reference	Clinical trials
Liraglutide	Inhibition of NLRP3 inflammasome and pyroptosis activation	NAFLD	(185–187)	NCT02147925 NCT03068065 NCT01399645 etc
Lactobacillus casei atc 393	Inhibition of NLRP3/Caspase-1/IL-1βpathway Reduction of NLRP3, caspase-1, IL-1β and IL-18	UC	(188)	null
β-acetylaminohexosidase (Amuc_2109)	Inhibition of TNF-α, IL-1β, IL-6 and NLRP3 expression	Colitis	(189)	null
AC-YVAD-CMK	Inhibition of caspase-1 Regulating the NF-κB pathway and P38 MAPK pathway	Acute gastric injury	(190)	null
Anakinra	IL-1RI inhibitor	Acute liver injury	(191)	null
Kinsenoside	Inhibition of NF-κB/NLRP3 signaling pathway	NASH	(192)	null

**Table 4:** Phytochemicals targeting on NLRP3 inflammasome in animal models of digestive diseases.

Phytochemicals	Molecular mechanisms	Experimental model	Reference
Celastrol	Inhibition of NF-κB pathway and pyroptosis Regulating oxidative stress level Block the cleavage of caspase-1	Liver damage colitis	(193, 194)
Sulforaphane	Suppression on oligomerization of TLR4 Blocking the transcription of NLRP3 and pro-IL-1β genes Promotion of autophagy	AP NAFLD	(195, 196)
Curcumin	Prevention of K^+^ efflux and Ca^2+^ influx Regulating P2X7R and NF-κB signaling pathway Impacting ASC oligomerization and spot formation	UC liver injury	(197, 198)
Formononetin	Inhibiting the NF-κB transcription signaling pathway	AIH, Colitis	(199, 200)
Salidroside	Regulating ROS and AMPK-dependent TXNIP/NLRP3 pathways	Acute liver injury NAFLD UC	(201–203)
Dihydromyricetin	Activating antioxidant pathways	liver injury	(204–206)
Dihydroartemisinin	Inhibition of the activation of NLRP3 inflammasome *via* p38 MAPK signaling	Colitis	(207)
Artesunate	Interrupting crosstalk of inflammatory and oxidative stress	Hepatic ischemia/reperfusion	(208)
Cardamonin	Inhibition of NLRP3 inflammasome activation *via* AhR/Nrf2/NQO1pathway	IBD	(209)
Pristimerin	Inhibition of NLRP3 and IL-1β secretion	Peritonitis	(210)
Chrysanthemum indicum	Inhibits NLRP3 inflammasome activation *via* regulating ASC phosphorylation	Peritonitis	(211)
Fraxinellone	Suppression NLRP3, PY-CARD, caspase-1, Il-18, IL-1β activation	Acute pancreatitis (AP)	(212)
C-phycocyanin	Inhibition of HMG-B1/NLRP3/NF-κB pathway	Gastric ulcers	(213)
Genipin	Inhibition of NLRP3 inflammasome activation	Acute gastric injury	(214)
Cryptotanshinone	Blocking Ca^2+^ signaling Reducing mitochondrial ROS	Non-alcoholic steatohepatitis (NASH)	(215)
Apocynin	Suppression NLRP3 inflammasome activation and NF-κB signaling pathway	Severe Acute Pancreatitis (SAP)	(216)
DSC	Inhibition of the activation of NF-κB, STAT3, and NLRP3 inflammasome	Acute Pancreatitis (AP)	(217)
Evodiamine	Regulation of NF-κB and NLRP3 inflammasome	Colitis	(218)

### 4.1 Small-molecule chemical drugs

It may be possible to treat digestive system diseases by directly targeting NLRP3 inflammasome. Several such inhibitors have been discovered to date. MCC950 is a potent and specific inhibitor of NLRP3 that acts on both canonical and non-canonical processes of NLRP3 inflammasome activation. MCC950 has the characteristics of high selectivity, strong inhibition effect, and good pharmacodynamic and pharmacokinetic properties (219). Previous studies have confirmed that MCC950 could modify the active conformation of NLRP3 (220) and target the NLRP3 ATP-hydrolysis motif in the NLRP3 NACHT domain, thus affecting the hydrolysis of ATP (221). MCC950 has been shown to play a therapeutic role in many digestive diseases involving pancreatic cancer, liver injury, HF, and UC (222). To be specific, in multiple cell lines derived from pancreatic cancer (SW1990, Panc1, & Panc10.05), MCC950 down-regulated NLRP3 expression and decreased cell activity to varying degrees. Interestingly, the effect was corresponded with the level of ACS (160). In addition, MCC950 could alleviate cholestatic liver injury (80) and CCL_4_-induced acute liver injury (161). MCC950 also reduced the levels of inflammatory factors and liver enzymes and improved HF in the NASH model (5). In a mouse model simulating UC, MCC950 reduced multiple pro-inflammatory factors released in the tissues of colon, relieved symptoms, and alleviated histopathological changes (162). OLT1177 is an active β-sulfonyl nitrile molecule and a selective NLRP3 inhibitor. OLT1177 can inhibit the activation of the NLRP3 inflammasome, reduce the activity of caspase-1, and inhibit the production of IL-1β and IL-18 (223). Oizumi T et al. reported that OLT1177 could block inflammasome assembly by preventing NLRP3 aggregation with ASC. As a result, OLT1177 effectively compensated for weight reduction, reduced disease activity index, histological score and inflammatory cytokines factors and alleviated DSS-induced colitis ultimately (163). Histone deacetylase 6 (HDAC6) inhibitor is recently reported as a NLRP3 small molecule inhibitor. Magupalli et al. proved that NLRP3 inflammasome activation depends on regulated ubiquitination (224) and engagement of the dynein adaptor HDAC6 to transport NLRP3 inflammasome to the microtubule-organizing center for activation in a ubiquitin-misfolded protein-like manner. HDAC6 deficiency compromises activation of the NLRP3 inflammasome, but not AIM2, NLR family CARD-containing protein 4 (NLRC4) and non-canonical inflammasomes (225). Moreover, selective HDAC6 inhibitors have therapeutic roles in IBD (164) and gastrointestinal cancers, including esophageal, gastric, colorectal, liver, and pancreatic cancer, as well as cholangiocarcinoma (226).

In addition to directly inhibiting the NLRP3 inflammasome, some pharmacological strategies can also be used to indirectly inhibit this inflammasome by taking advantage of its complex signaling cascade, including transcription and oligomerization inhibition, autophagy modulation, GSDMD cleavage inhibition, etc.

Blocking the NF-κB-mediated transcriptional efforts can significantly inhibit NLRP3 oligomerization. Kanak MA et al. showed that withaferin A (WA), a steroidal lactone derived from the Withania somnifera plant, could effectively reduce immune cell infiltration, acinar cell death and sustained ER stress response in CP by blocking the activation of the NLRP3 inflammasome through NF-κB pathway (165). It has been found that ursolic acid (UA) reduced the expression of the NLRP3 inflammasome and release of pro-inflammatory cytokines by inhibiting the NF-κB signaling pathway, thus showing significant inhibit proliferation in both gastric tumour models and human gastric carcinoma cells (37). Dapagliflozin (DPZ) is a drug employed in the treatment of diabetes. Recently, it has been found that DPZ could inhibit the activation step of NLRP3 inflammasome by regulating the NF-κB/Adenosine 5’-monophosphate (AMP)-activated protein kinase (AMPK) interplay and interrupting NLRP3/caspase-1 signaling. DPZ exhibits beneficial effects in UC models induced by acetic acid (176). The histological and macroscopic characteristics of colon tissue were improved, and the survival time was prolonged. DPZ could also improve hepatic lipid accumulation and fibrotic response in steatohepatitis with diabetes mellitus (177).

The use of ROS inhibition as a pharmacological target is beneficial for blocking the assembly and activation of NLRP3 inflammasome. Angiotensin II (Ang II) has been proved to increase recombinant nicotinamide adenine dinucleotide phosphate oxidase 4 (NOX4) expression and ROS and leads to the NLRP3 inflammasome activation. Angiotensin (1–7) has a reverse regulatory effect on Ang II and can regulate redox equilibrium, thereby improving HF (172). Simvastatin (SIM) can increase the antioxidant level of glutathione (GSH) and super oxide dismutase (SOD) in a dose-dependent manner, thereby reducing the IL-1β, caspase-1, NLRP3, tumor necrosis factor-α (TNF-α), malondialdehyde (MDA), and cyclooxygenase-2 (COX-2) content in the colon, and improving the mucosal histological score of UC (182).

Another critical pharmacological strategy with beneficial effects in digestive disorders is to modulate autophagy. Autophagy is vital self-protective process for recycling and removal of damaged proteins and organelles, has been proved to inhibit the activation process of the NLRP3 inflammasome. F240B is a new synthetic 4-hydroxy auxarconjugatin B (4-HAB) analog. 4-HAB is an autophagy inducer (227). It has been found that F240B could inhibit ASC oligomerization and pro-IL-1β expression in an autophagy-dependent manner and accelerated the degradation of NLRP3 and IL-1β. Additionally, F240B showed an anti-inflammatory effect in peritonitis mice model (166). GL-V9 (5-hydroxy-8-methoxy-2-phenyl-7-(4-(pyrrolidine-1-yl) butoxy)4 H-chromen-4-one) is a new synthesized flavonoid derivative. GL-V9 induces autophagy by activating AMPK, thereby blocking NLRP3 inflammasome activation, and exhibits therapeutic potential in liver cancer model (167, 168). A recent study has shown that GL-V9 had a significant anti-inflammatory effect in a colorectal cancer model associated with colitis. Gl-v9 also reduced intestinal mucosal injury and relieved the severity of enteritis by degrading NLRP3 inflammasome (169). The dipeptidyl peptidase-4 inhibitor saxagliptin is a selective inhibitor of the enzyme. It was reported that saxagliptin could induce autophagy *via* AMPK/mTOR and inhibit NLRP3, ASC, NF-κB, caspase-1, IL-1β expression, and decrease gastric pathological symptoms, including ulcer area, ulcer index score, and histopathologic abnormalities (180). Dehydroepiandrosterone is a vital cholesterol metabolic intermediate and shows protection against DSS-induced colitis both *in vivo* and *in vitro* (174). Notably, a recent study reported that Dehydroepiandrosterone induced autophagy *via* G protein-coupled estrogen receptorgper (GPER) activation and inhibited NF-κB signaling pathway, leading to decreasing the expression of NLRP3 inflammasome components (228).

Of note, preclinical studies have reported the beneficial effects arising from inhibiting GSDMD cleavage and blocking the proinflammatory cytokine cascade. Rabeprazole could reduce inflammatory responses through inhibition of GSDMD-mediated pyroptosis in gastric epithelial cells and reducing the maturation and secretion of IL-1β and IL-18 (29). Metformin could decrease pro-inflammatory factors, protect against intestinal ischemia-reperfusion injury in a TXNIP-NLRP3-GSDMD-dependent manner (181).

### 4.2 Biologics

Multiple biological agents, including both inhibitors of NLRP3 activation and blockers of inflammasome signaling, have been demonstrated to have beneficial effects in studies of disorders of the digestive system.

Liraglutide, a glucagon-like peptide-1 (GLP-1) analogue that has become the first-line treatment for type 2 diabetes mellitus (T2DM), is found beneficial in digestive diseases. Experiments show that liraglutide could suppress NLRP3 inflammasome-induced hepatocyte pyroptosis *via* mitophagy to slow the progression of NASH (185). Clinical studies have demonstrated that liraglutide can effectively reduce the visceral adipose tissue and the proton density fat fraction estimated by magnetic resonance imaging in patients with T2DM and NAFLD (186).

AC-YVAD-CMK can selective block caspase-1, which can suppress the IL-1β production (229). Besides the inhibition of caspase-1, AC-YVAD-CMK can also alleviate the acute gastric injury by regulating the NF-κB pathway and P38 mitogen-activated protein kinase (MAPK) pathway, and inhibiting NLRP3 inflammasome activation indirectly (190). Kinsenoside (KD), naturally isolated from Anoectochilus roxburghii, could also inhibit NF-κB/NLRP3 signaling pathway and alleviate experimental NASH (192).

Anakinra is a recombinant interleukin-1 receptor type I (IL-1RI) inhibitor (230). Studies have shown that Anakinra can reduce the severity of liver injury induced by D-galactoamine (D-GALn) and LPS, which is manifested by the reduction of AST and ALT (191). IL-1β, the most widely recognized downstream mediator of the NLRP3 inflammasome. Anti-IL-1 biologic drugs could represent innovative therapeutical options for the management of digestive system diseases. However, it is worth noting that IL-1β production can be mediated by other inflammasomes or by inflammasome-independent pathways. Therefore, inhibitors aimed at IL-1β may result in unintentional immunosuppressive effects. In this respect, further investigations are needed to better understand the role of NLRP3-dependent IL-1β release.

### 4.3 Phytochemicals

According to the results of *in vitro* and *in vivo* studies, natural products can also act as inhibitors of NLRP3 inflammasome*in vitroin vivo*, and have clinical value once the efficacy and safety are verified.

Inhibition of upstream ROS/TXNIP/NLRP3 interaction, has also been found to alleviate digestive diseases. Salidroside (SAL) is a naturally occurring phenolic compound found in Rhodiola Sachalinensis. SAL reduced obesity, abnormal blood glucose and liver lipid deposition in NAFLD mouse model by regulating ROS and AMPK-dependent TXNIP/NLRP3 pathways (201). Moreover, SAL also showed therapeutic potential in UC and CCl_4_-induced liver injury models (202, 203). Dihydromyricetin (DHM) is a polyphenol which isolates from Ampelopsis grossedentata. DHM through activation antioxidant pathways and reducing inflammasome expression, significantly improved liver injury induced by LPS, decreased liver enzyme levels, reduced histopathological changes and ultrastructure (204). DHM also showed similar liver-protecting and lowering enzymes effect in CCL_4_-induced liver injury model (205). In addition, DHM led to the inhibition of NLRP3, caspase-1, IL-1β and IL-18 expression as well as the damage of intestinal mucosa induced by pyroptosis in LPS-induced ileum injury model (206).

Numerous phytochemicals are known to protect against digestive disorders by blocking both of the steps that lead to the activation and priming of the inflammasome. Formononetin (FMN) is a major flavonoid component extracted from Astragalus membranaceus. Liu G et al. found that FMN inhibits the NF-κB transcription signaling pathway, and inhibits the activation of the NLRP3 inflammasome in the liver tissue of ConA-induced AIH model, significantly reducing the levels of proinflammatory cytokines in mouse serum and liver tissue, inhibiting hepatocyte apoptosis and alleviating liver tissue damage (199). In addition, FMN also shows a protective effect on the colonic mucosa (200). Sulforaphane (SFN), a dietary phytochemical, is a significant member of isothiocyanates. SFN can suppress on oligomerization of TLR4, promote autophagy through the AMPK signaling pathway, and block expression of the NLRP3 gene and pro-IL-1β, but not AIM2 inflammasome (231, 232). At present, the research of SFN in digestive system diseases is mainly concentrated in AP and NAFLD (195, 233). Curcumin, the main polyphenol contained in turmeric root (Curcuma longa), is popular as dietary supplements and topical medications for treating inflammatory conditions (234). The inhibiting effect of curcumin in NLRP3 inflammasome and IL-1β depends on preventing K^+^ efflux and Ca^2+^ influx, reversing the activation of P2X7R, regulating NF-κB signaling pathway, interfering with the effective spatial arrangement of mitochondria, impacting ASC oligomerization and spot formation (235, 236). Curcumin can alleviate DSS-induced colitis (237) and aflatoxin B1 (AFB1)-induced liver injury (197). It has been demonstrated that Celastrol, derived from the medicinal plant tripterygium wilfordii, is capable of significantly reducing inflammation in various digestive disorders. Celastrol inhibits NLRP3 inflammasome activation *via* suppressing NF-κB pathway as well as OS level, significantly reducing the secretion of IL-1β and IL-18. Celastrol can also selectively block the cleavage of caspase-1 and inhibit pyroptosis (193, 238). Celastrol has been found to improve the histopathological features of colitis, protect intestinal mucosal homeostasis (193), and reduce propionibacterium acnes/LPS-induced liver injury (194).

### 4.4 Discussions

The NLRP3 inflammasome is a promising drug target. This pathway appears to be central to a broad range of indications as evidenced by the variety of indications for which it is implicated. In clinical and preclinical trials, small-molecule chemical drugs, biologics, and phytochemicals have been shown to block the transcription, oligomerization, and activation of the NLRP3 inflammasome. These studies may provide innovative therapeutic options for digestion problems. Therein, some medications work more effectively when they are combined with others. For example, empagliflozin and metformin combination strongly activated AMPK phosphorylation and suppressed mTOR expression leading to a robust inhibitory effect on NLRP3 inflammasome assembly and caspase-1 cleavage. The combined administration of both drugs bypassed the decreased efficacy of their individual administration and exhibited greater protective and ameliorative effects on ulcerative colitis (183). rosuvastatin and lactobacillus combination attenuated the inflammatory response by inhibiting NLRP3 inflammasome assembly, and significantly suppressed the DSS/high-fat diet-induced IBD (184). Compared to one medication only, the combination of Rosuvastatin with Lactobacillus provides further protection by correcting dysbiosis. Of interest, although IL-1β blockage presents an attractive avenue to treat inflammatory disease, IL-1β production can be mediated by other inflammasomes or by inflammasome-independent pathways. Inhibitors aimed at IL-1β can result in unintentional immunosuppressive effects. Therefore, direct targeting of NLRP3 activation may be more effective, specific, and cost-effective. However, there remain considerable challenges for the use of NLRP3 blockade. At present, most of the pharmacological entities have been tested only in animal models and are not approved for clinical use (239). A Phase II clinical trial of MCC950 for rheumatoid arthritis was suspended due to hepatic toxicity (51). On these bases, patient-centered long-term randomized clinical trials with a larger sample size are urgently needed to assess the therapeutic effects, integrated safety, pharmacokinetic properties, stability, oral bioavailability, and off-target immunosuppressive effects of NLRP3 inhibitors. A direct structure-induced inhibitor with improved specificity and efficacy should also be developed using the advantage of the existing NLRP3 structure. Continued profiling, refinement and repurposing of these specific NLRP3 inhibitors will boost future clinical translation, epitomizing the use of precision medicine in NLRP3 inflammasome related digestive disorders. Given that the good safeties and broad biological activities of phytochemicals, future studies may confer additional insights on these potential inhibitors and bring them to clinic.

## 5 Prospect

One of the most important components of the innate immune system is the NLRP3 inflammasome, which is not only one of the vital components of pattern recognition receptors, but also a critical link in initiating the downstream inflammatory cascade reaction, which has become one of the research hotspots in recent years. As a key regulatory factor of inflammatory response, its expression level and activation intensity play an important role in the occurrence and development of digestive system diseases. The multilevel fine regulation of NLRP3 inflammasome pathway contributes to the treatment of digestive diseases and facilitates the research and development of precision therapeutic targets. Despite the current results of NLRP3 inflammasome studies, many questions remain to be answered. The inflammasome cross-links with other signaling pathways, and existing drugs are difficult to avoid targeting molecules other than the NLRP3 signaling pathway. Therefore, in the future inhibitor screening process, we should identify the range of possible drug targets and try to avoid potential side effects. The benefits of these inhibitors still need to be tested in large, multicenter, prospective randomized controlled clinical trials. At the same time, our understanding of the role of NLRP3 inflammasome in the digestive system is still limited, given the complexity of diseases and the diversity of NLRP3 inflammasome regulatory networks. To understand the exact role of NLRP3 inflammasome in digestive disorders, more extensive research and experiments are required.

## Author contributions

Designed, written, reviewed and illustrated by RQ and YL. RQ and YL contributed equally to this work. Critically revised and edited by XD. WL puts forward some valuable suggestions for this paper. RQ, YL, and WL are the co-corresponding authors. All authors contributed to the article and approved the submitted version.

## Funding

This work was supported by Ministry of Science and Technology of the People’s Republic of China (Grant numbers: No. 2018YFC1705700). The name of fund is National Key Research and Development Program of China.

## Acknowledgments

We are grateful to the Yingke Qianxin team for helping us to search the literature.

## Conflict of interest

The authors declare that the research was conducted in the absence of any commercial or financial relationships that could be construed as a potential conflict of interest.

## Publisher’s note

All claims expressed in this article are solely those of the authors and do not necessarily represent those of their affiliated organizations, or those of the publisher, the editors and the reviewers. Any product that may be evaluated in this article, or claim that may be made by its manufacturer, is not guaranteed or endorsed by the publisher.
